# Comparison of Diabetes Medications Used by Adults With Commercial Insurance vs Medicare Advantage, 2016 to 2019

**DOI:** 10.1001/jamanetworkopen.2020.35792

**Published:** 2021-02-01

**Authors:** Rozalina G. McCoy, Holly K. Van Houten, Yihong Deng, Pinar Karaca Mandic, Joseph S. Ross, Victor M. Montori, Nilay D. Shah

**Affiliations:** 1 Division of Community Internal Medicine, Department of Medicine, Mayo Clinic, Rochester, Minnesota; 2 Division of Health Care Policy and Research, Department of Health Sciences Research, Mayo Clinic, Rochester, Minnesota; 3 Mayo Clinic Robert D. and Patricia E. Kern Center for the Science of Health Care Delivery, Rochester, Minnesota; 4 Department of Finance and Medical Industry Leadership Institute, Carlson School of Management, University of Minnesota, Minneapolis; 5 National Bureau of Economic Research, Cambridge, Massachusetts; 6 National Clinician Scholars Program, Department of Internal Medicine, Yale School of Medicine, New Haven, Connecticut; 7 Section of General Internal Medicine, Department of Internal Medicine, Yale School of Medicine, New Haven, Connecticut; 8 Department of Health Policy and Management, Yale School of Public Health, New Haven, Connecticut; 9 Center for Outcomes Research and Evaluation, Yale–New Haven Hospital, New Haven, Connecticut; 10 Division of Endocrinology, Diabetes, Metabolism, and Nutrition, Department of Medicine, Mayo Clinic, Rochester, Minnesota; 11 Knowledge Evaluation Research Unit, Rochester, Minnesota; 12 OptumLabs, Cambridge, Massachusetts

## Abstract

**Importance:**

Glucagonlike peptide-1 receptor agonists (GLP-1RA), sodium-glucose cotransporter-2 inhibitors (SGLT2i), and dipeptidyl peptidase-4 inhibitors (DPP-4i) are associated with low rates of hypoglycemia, and postmarketing trials of GLP-1RA and SGLT2i demonstrated that these medications improved cardiovascular and kidney outcomes.

**Objective:**

To compare trends in initiation of treatment with GLP-1RA, SGLT2i, and DPP-4i by older adults with type 2 diabetes insured by Medicare Advantage vs commercial health plans.

**Design, Setting, and Participants:**

This retrospective cohort study used administrative claims data from a deidentified database of commercially insured and Medicare Advantage beneficiaries. Adults aged 58 to 66 years with type 2 diabetes who filled any medication prescription to lower glucose levels from January 1, 2016, to December 31, 2019, were compared between groups.

**Exposure:**

Enrollment in a Medicare Advantage or commercial health insurance plan.

**Main Outcomes and Measures:**

The odds of initiating GLP-1RA, SGLT2i, and DPP-4i treatment were examined for Medicare Advantage vs commercial insurance beneficiaries using 3 separate logistic regression models adjusted for year and demographic and clinical factors. These models were used to calculate adjusted annual rates of medication initiation by health plan.

**Results:**

A total of 382 574 adults with pharmacologically treated type 2 diabetes (52.9% men; mean [SD] age, 62.4 [2.7] years) were identified, including 172 180 Medicare Advantage and 210 394 commercial beneficiaries. From 2016 to 2019, adjusted rates of initiation of GLP-1RA, SGLT2i, and DPP-4i treatment increased among all beneficiaries, from 2.14% to 20.02% for GLP-1RA among commercial insurance beneficiaries and from 1.50% to 11.44% among Medicare Advantage beneficiaries; from 2.74% to 18.15% for SGLT2i among commercial insurance beneficiaries and from 1.57% to 8.51% among Medicare Advantage beneficiaries; and from 3.30% to 11.71% for DPP-4i among commercial insurance beneficiaries and from 2.44% to 7.68% among Medicare Advantage beneficiaries. Initiation rates for all 3 drug classes were consistently lower among Medicare Advantage than among commercial insurance beneficiaries. Within each calendar year, the odds of initiating GLP-1RA treatment ranged from 0.28 (95% CI, 0.26-0.29) to 0.70 (95% CI, 0.65-0.75) for Medicare Advantage and commercial insurance beneficiaries, respectively; SGLT2i, from 0.21 (95% CI, 0.20-0.22) to 0.57 (95% CI, 0.53-0.61), respectively; and DPP-4i, from 0.37 (95% CI, 0.34-0.39) to 0.73 (95% CI, 0.69-0.78), respectively (*P* < .001 for all). The odds of starting GLP-1RA and SGLT2i increased with income; for an income of $200 000 and higher vs less than $40 000, the odds ratio for GLP-1RA was 1.23 (95% CI, 1.15-1.32) and for SGLT2i was 1.16 (95% CI, 1.09-1.24).

**Conclusions and Relevance:**

These findings suggest that Medicare Advantage beneficiaries may be less likely than commercially insured beneficiaries to be treated with newer medications to lower glucose levels, with greater disparities among lower-income patients. Better understanding of nonclinical factors contributing to treatment decisions and efforts to promote greater equity in diabetes management appear to be needed.

## Key Points

**Question:**

Were there differences in the initiation of treatment with newer medications to lower glucose levels between patients with type 2 diabetes insured by Medicare Advantage and those insured by commercial health plans from 2016 to 2019?

**Findings:**

In this cohort study of 382 574 adults with type 2 diabetes aged 58 to 66 years, rates of initiation of glucagonlike peptide-1 receptor agonists, sodium-glucose cotransporter-2 inhibitors, and dipeptidyl peptidase-4 inhibitors were lower among Medicare Advantage enrollees than among commercial health insurance plan enrollees.

**Meaning:**

These findings suggest that a better understanding of nonclinical factors contributing to treatment selection and efforts to promote equity in diabetes management are needed.

## Teaser

This cohort study compares trends in initiation of treatment with glucagonlike peptide-1 receptor agonists, sodium-glucose cotransporter-2 inhibitors, and dipeptidyl peptidase-4 inhibitors by older adults with type 2 diabetes insured by Medicare Advantage vs commercial health plans.

## Introduction

Type 2 diabetes is one of the most common and serious chronic health conditions in the US,^1^ with a disproportionately high burden of disease among older adults. More than 1 in 4 older adults have diabetes (26.8% of US residents 65 years or older),^1^ and nearly 90% of older adults with a diagnosis of diabetes are taking medications to lower glucose levels.^2^ Optimal diabetes management is predicated on controlling hyperglycemia, avoiding hypoglycemia, and using medications associated with the greatest benefit and the least risk of harm when contextualized against each patient’s preferences and situation. Although metformin is consistently recommended as the first-line drug in the management of type 2 diabetes, clinical guidelines advise that the choice of second-line therapy to be used when metformin is either no longer sufficient or is not tolerated is informed by the presence of key comorbidities (ie, cardiovascular disease, heart failure, and nephropathy or chronic kidney disease), risk for hypoglycemia, and considerations of affordability.^3,4,5,6,7^ Three classes of medications to lower glucose levels were introduced into practice during the past 2 decades. Glucagonlike peptide-1 receptor agonists (GLP-1RA), sodium-glucose cotransporter-2 inhibitors (SGLT2i), and dipeptidyl peptidase-4 inhibitors (DPP-4i) are associated with low rates of hypoglycemia, and postmarketing trials of GLP-1RA and SGLT2i also demonstrated that these drugs improved cardiovascular, kidney, and mortality outcomes.^8,9,10,11,12,13,14,15,16,17,18,19,20,21^

Medicare Advantage, also called Medicare Part C, is an all-in-one private alternative to traditional (fee-for-service) Medicare, a national health insurance program for retirees, people with disabilities, and patients with end-stage kidney disease requiring dialysis. Medicare Advantage plans are funded by capitated payments from the federal government to private insurance companies to provide Medicare beneficiaries with health insurance that approximates commercial insurance plans for the population younger than 65 years. In addition to covering all services reimbursed by traditional Medicare, 90% of Medicare Advantage plans include an integrated prescription drug benefit, and many plans offer coverage for vision, hearing, dental, and wellness programs.^22^ In recent years, enrollment in Medicare Advantage plans has been steadily increasing by nearly 9% annually.^22^ Currently, 36% of all Medicare beneficiaries (24.1 million of 67.7 million people) are enrolled in Medicare Advantage plans, with as many as 51% expected to be enrolled by 2030.^22^ The increasing popularity of Medicare Advantage plans calls for better understanding of the use of prescription drugs, particularly by patients with common chronic health conditions such as diabetes.

Data regarding diabetes management for people with Medicare Advantage vs commercial health insurance coverage are scarce. McCoy et al^23^ previously found low rates of early adoption of SGLT2i among enrollees in Medicare Advantage plans compared with commercial plans. With greater experience using SGLT2i medications and emerging evidence supporting their preferred use in the context of heart failure and nephropathy, prescribing practices may have changed. Moreover, whether use patterns of other potentially preferred medications to lower glucose levels, specifically GLP-1RA, which would be favored in the presence of cardiovascular and kidney disease, differ between Medicare Advantage and commercial enrollees is unknown. To address this knowledge gap with important implications for health policy and health benefit design, we examined the trends in initiation of GLP-1RA, SGLT2i, and DPP-4i treatment among Medicare Advantage and commercial health plan beneficiaries with type 2 diabetes aged 58 to 66 years from 2016 to 2019, hypothesizing that despite the emerging data, these medications will continue to be used less frequently by Medicare Advantage enrollees.

## Methods

### Study Design

This retrospective cohort study assessed deidentified administrative claims data from OptumLabs Data Warehouse, which includes medical and pharmacy claims and enrollment records for commercial insurance plan and Medicare Advantage enrollees.^24,25,26^ The OptumLabs Data Warehouse contains longitudinal health information on enrollees, representing a diverse mixture of ages, races/ethnicities, and geographic regions across the US. This study was exempt from review by the Mayo Clinic institutional review board because it involved research on preexisting and deidentified data. This study followed the Strengthening the Reporting of Observational Studies in Epidemiology (STROBE) guideline.^27^

### Study Population

We included all enrollees with type 2 diabetes aged 58 to 66 years who filled any medication prescription to lower glucose levels (eTable 1 in the Supplement) from January 1, 2016, to December 31, 2019. This age range was selected empirically by requiring that at least 1% of beneficiaries in each 1-year age subset have both Medicare Advantage and commercial insurance coverage to minimize bias when comparing between health plans (eFigure in the Supplement). For patients in the GLP-1RA, SGLT2i, and DPP-4i cohorts, the index date was set at the first prescription fill for a drug in each class that was preceded by a 12-month period of uninterrupted health insurance coverage without a prescription fill for any drug in the same therapeutic class. For all other patients, the index date was set at the first prescription fill of any drug that was preceded by 12 months of uninterrupted health insurance coverage. Diabetes was ascertained using Healthcare Effectiveness Data and Information Set claims–computable criteria^28^ applied to the 12-month period preceding the index date.

Diabetes type was ascertained based on codes from the *International Classification of Diseases, Ninth Revision, Clinical Modification* (*ICD-9-CM*), and the *International Statistical Classification of Diseases, Tenth Revision, Clinical Modification (ICD-10-CM)*, and prescriptions for medications filled during the 12 months preceding the index date, consistent with previously described methods.^23,29,30^ Patients with type 1 diabetes were excluded. In brief, type 1 diabetes was assumed for patients who had (1) more type 1 than type 2 diabetes diagnosis codes on evaluation and management visit claims and insulin claims or (2) an equal number of type 1 and type 2 diabetes diagnosis codes, bolus insulin claims, and no sulfonylurea claims. Codes indicative of type 1 diabetes included *ICD-9-CM* 250.x1 and 250.x3 and *ICD-10-CM* codes E10.xxx and O24.0xx. Codes indicative of type 2 diabetes included *ICD-9-CM* 250.x0 and 250.x2 and *ICD-10-CM* E11.xxx and O24.1xx. Evaluation and management visits were identified by *Current Procedural Terminology* codes 99201 to 99499.

### Outcomes

First starts (initiation) of GLP-1RA, SGLT2i, and DPP-4i treatment were independently ascertained from pharmacy prescription fills, requiring no fills for the same drug class during the preceding 12-month period (but allowing fills for any other drug class, including the other classes of interest). Medication use was classified as first line if there were no prescription fills for any classes of glucose-lowering medications in the preceding 12 months. The 3 outcomes were examined separately and were not mutually exclusive. These medication classes have the same formulary designations in Medicare Advantage and commercial insurance plans.^31^

### Other Covariates

Explanatory variables included enrollment in a Medicare Advantage vs commercial health plan. Patient characteristics ascertained at the time of the index date included age, sex, race/ethnicity, US region of residency, and annual household income. Clinical variables included specialty of the physician prescribing the index medication (ascertained based on the National Provider Identifier or the Drug Enforcement Administration number on the pharmacy claim); therapeutic classes of medications with prescriptions filled within 120 days before the index date categorized according to eTable 1 in the Supplement, with combination medications counted toward each component therapeutic class and patients without any baseline medications categorized as having none; whether the index medication was used as first- or second-line treatment; the calendar year of initiation of the index drug; and comorbidities ascertained from all claims during the 12 months preceding the index date. For comorbidities, we considered the total count of diabetes-related complications ascertained using the Diabetes Complications Severity Index (retinopathy, nephropathy, neuropathy, cerebrovascular disease, cardiovascular disease, and peripheral vascular disease)^32^ and the individual presence of myocardial infarction, cerebrovascular disease, heart failure, nephropathy, retinopathy, neuropathy, peripheral vascular disease, dementia, chronic obstructive pulmonary disease, cirrhosis, cancer (except for skin cancer), and severe hypoglycemia and hyperglycemia, both of which were ascertained from primary diagnoses on emergency department or hospital claims only. Diagnosis codes used to define these comorbidities are detailed in eTable 2 in the Supplement. These covariates were selected based on their clinical relevance and prior literature demonstrating their potential association with choice of therapy to lower glucose levels.

### Statistical Analysis

Characteristics of patients treated with GLP-1RA, SGLT2i, DPP-4i, and all pharmacological therapies are reported as frequencies with percentages for categorical data and means with SDs or medians with interquartile ranges (IQRs) for continuous variables. Data were compared using unpaired *t* tests for continuous variables and χ^2^ tests for categorical variables. Univariate logistic regression models compared those who initiated GLP-1RA, SGLT2i, and DPP-4i treatment with those who did not initiate these treatments.

Three separate multivariate logistic regression models were used to assess factors associated with GLP-1RA, SGLT2i, and DPP-4i initiation compared with no initiation, with results presented as odds ratios (ORs) and 95% CIs. Model covariates included the covariates detailed above, with missing data included as a separate category (unknown), and interaction terms between year and health plan. These models were then used to calculate predicted probabilities of initiation of GLP-1RA, SGLT2i, and DPP-4i treatment in each calendar year by health plan among all patients and in subgroups of patients with myocardial infarction, cerebrovascular disease, stroke, and nephropathy. Two-sided *P* < .05 indicated statistical significance. All analyses were conducted using SAS Enterprise Guide, version 9.4 (SAS Institute).

## Results

We identified 382 574 adults aged 58 to 66 years with prescriptions for pharmacological treatment for type 2 diabetes (52.9% men and 47.1% women; mean [SD] age, 62.4 [2.7] years), including 172 180 (45.0%) with Medicare Advantage and 210 394 (55.0%) with commercial insurance. The mean (SD) age of Medicare Advantage beneficiaries was 63.4 (2.7) years; 53.7% were women, 49.8% were White, and 40.2% had an annual household income of less than $40 000 (Table 1). In contrast, patients with commercial insurance were younger, with a mean (SD) age of 61.5 (2.4) years; 41.7% were women, 66.0% were White, and 18.6% had an annual household income of less than $40 000. Medicare Advantage beneficiaries had a greater burden of diabetes-related complications, with 39.6% of Medicare Advantage and 16.4% of commercial beneficiaries having 2 or more complications.

**Table 1.** Study Population of Pharmacologically Treated Older Adults With Type 2 Diabetes, 2016 to 2019

Characteristic	Patient group (N = 382 574)^a^		*P* value
	Commercial	Medicare Advantage	
All patients	210 394 (55.0)	172 180 (45.0)	NA
Age, mean (SD), y	61.5 (2.4)	63.4 (2.7)	<.001
Sex			
Female	87 740 (41.7)	92 402 (53.7)	<.001
Male	122 654 (58.3)	79 778 (46.3)	
Race/ethnicity			
White	138 947 (66.0)	85 795 (49.8)	<.001
Black	23 922 (11.4)	37 106 (21.6)	
Hispanic	25 702 (12.2)	19 960 (11.6)	
Asian	7555 (3.6)	3460 (2.0)	
Unknown	14 268 (6.8)	25 859 (15.0)	
Annual household income			
<$40 000	39 208 (18.6)	69 168 (40.2)	<.001
$40 000-$74 999	52 656 (25.0)	39 060 (22.7)	
$75 000-$124 999	63 221 (30.0)	23 891 (13.9)	
$125 000-$199 999	27 454 (13.0)	5104 (3.0)	
≥200 000	13 217 (6.3)	1256 (0.7)	
Unknown	14 638 (7.0)	33 701 (19.6)	
US region			
Midwest	59 342 (28.2)	34 164 (19.8)	<.001
Northeast	16 394 (7.8)	22 629 (13.1)	
South	103 485 (49.2)	103 511 (60.1)	
West	31 173 (14.8)	11 876 (6.9)	
Baseline drugs			
GLP-1RA	16 577 (7.9)	10 414 (6.0)	<.001
SGLT2i	19 013 (9.0)	8178 (4.7)	<.001
DPP-4i	23 750 (11.3)	17 306 (10.1)	<.001
Metformin	111 326 (52.9)	91 645 (53.2)	.05
Sulfonylurea	44 881 (21.3)	40 355 (23.4)	<.001
Thiazolidinediones	10 900 (5.2)	7471 (4.3)	<.001
Basal insulin	27 206 (12.9)	36 185 (21.0)	<.001
Bolus insulin	13 238 (6.3)	19 515 (11.3)	<.001
Other	391 (0.2)	377 (0.2)	.02
None	68 100 (32.4)	42 169 (24.5)	<.001
No. of diabetes complications			
Mean (SD)	0.7 (1.0)	1.4 (1.3)	<.001
Median (IQR)	0 (0-1)	1 (0-2)	
No. of diabetes complications			
0	117 022 (55.6)	53 037 (30.8)	<.001
1	58 843 (28.0)	50 916 (29.6)	
2	22 671 (10.8)	34 011 (19.8)	
3	8295 (3.9)	19 971 (11.6)	
≥4	3563 (1.7)	14 245 (8.3)	
Comorbidities			
Myocardial infarction	5621 (2.7)	8794 (5.1)	<.001
Cerebrovascular disease	10 820 (5.1)	22 030 (12.8)	<.001
Heart failure	8226 (3.9)	23 380 (13.6)	<.001
Nephropathy	22 823 (10.8)	38 479 (22.3)	<.001
Retinopathy	20 128 (9.6)	28 576 (16.6)	<.001
Neuropathy	34 023 (16.2)	59 485 (34.5)	<.001
Peripheral vascular disease	14 775 (7.0)	32 414 (18.8)	<.001
Dementia	397 (0.2)	3111 (1.8)	<.001
COPD	15 415 (7.3)	36 804 (21.4)	<.001
Cancer	13 312 (6.3)	13 485 (7.8)	<.001
Cirrhosis	1942 (0.9)	3552 (2.1)	<.001
Severe hyperglycemia	750 (0.4)	1156 (0.7)	<.001
Severe hypoglycemia	530 (0.3)	1836 (1.1)	<.001
Prescriber specialty			
Endocrinology	17 230 (8.2)	7850 (4.6)	<.001
Family medicine	88 691 (42.2)	57 074 (33.1)	
Internal medicine	60 627 (28.8)	45 125 (26.2)	
Cardiology	1228 (0.6)	1386 (0.8)	
Other	34 726 (16.5)	4878 (2.8)	
Unknown	7892 (3.8)	55 867 (32.4)	

Abbreviations: COPD, chronic obstructive pulmonary disease; DPP-4i, dipeptidyl peptidase-4 inhibitors; GLP-1RA, glucagonlike peptide-1 receptor agonists; IQR, interquartile range; NA, not applicable; SGLT2i, sodium-glucose cotransporte-2 inhibitors.

^a^ Includes older adults aged 58 to 66 years with type 2 diabetes at the time of their first prescription fill of a drug to lower glucose levels. Unless otherwise indicated, data are expressed as number (percentage) of patients. Percentages have been rounded and may not total 100.

Overall, 6.6% of Medicare Advantage beneficiaries and 9.7% of commercial beneficiaries started GLP-1RA treatment during the study period; 4.7% of Medicare Advantage beneficiaries and 9.8% of commercial beneficiaries started SGLT2i treatment; and 5.7% of Medicare Advantage beneficiaries and 7.2% of commercial beneficiaries started DPP-4i treatment. Characteristics of patients starting treatment with these 3 drug classes are detailed in eTables 3 to 5 in the Supplement.

**Figure 1.**
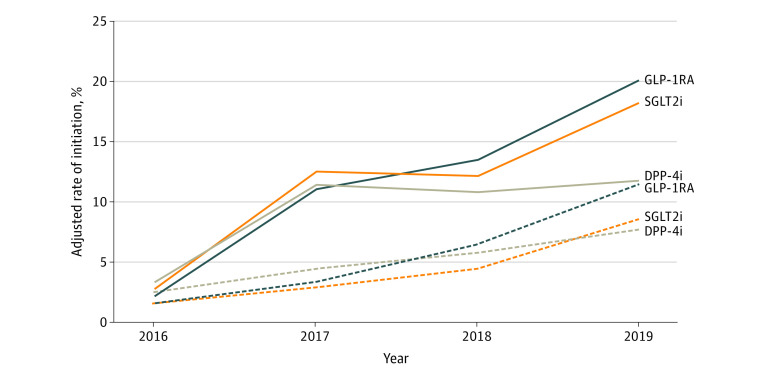
Trends in Use of Medication to Lower Glucose Levels by Health Plan Adjusted proportions (ie, predicted probability) of older adults with type 2 diabetes with commercial (solid lines) and Medicare Advantage (dashed lines) health insurance coverage who initiated use of a glucagonlike peptide-1 receptor agonist (GLP-1RA), sodium-glucose cotransporter-2 inhibitor (SGLT2i), or dipeptidyl peptidase-4 inhibitor (DPP-4i) from 2016 to 2019.

As shown in Table 2, Table 3, and Figure 1, adjusted rates of GLP-1RA initiation increased steadily from 2016 to 2019, from 2.14% to 20.02% among commercial beneficiaries (OR, 11.44; 95% CI, 10.92-11.98) and from 1.50% to 11.44% among Medicare Advantage beneficiaries (OR, 8.46; 95% CI, 7.87-9.11). Similarly, adjusted rates of SGLT2i initiation increased from 2.74% to 18.15% among commercial beneficiaries (OR, 7.87; 95% CI, 7.53-8.23) and from 1.57% to 8.51% among Medicare Advantage beneficiaries (OR, 5.83; 95% CI, 5.39-6.30). Initiation rates for DPP-4i increased less steeply, from 3.30% to 11.71% among commercial beneficiaries (OR, 3.89; 95% CI, 3.70-4.09) and from 2.44% to 7.68% among Medicare Advantage beneficiaries (OR, 3.33; 95% CI, 3.10-3.57). Within each calendar year, the odds of medication initiation were significantly lower for Medicare Advantage beneficiaries than for commercial beneficiaries, ranging from ORs of 0.28 (95% CI, 0.26-0.29) to 0.70 (95% CI, 0.65-0.75) for GLP-1RA, 0.21 (95% CI, 0.20-0.22) to 0.57 (95% CI, 0.53-0.61) for SGLT2i, and 0.37 (95% CI, 0.34-0.39) to 0.73 (95% CI, 0.69-0.78) for DPP-4i.

**Table 2.** Factors Associated With Starting New Glucose-Lowering Medications by Older Adults With Type 2 Diabetes, 2016 to 2019^a^

Factor	GLP-1RA use		SGLT2i use		DPP-4i use	
	OR (95% CI)	*P* value	OR (95% CI)	*P* value	OR (95% CI)	*P* value
Year of treatment initiation						
Medicare Advantage vs commercial insurance						
2016	0.70 (0.65-0.75)	<.001	0.57 (0.53-0.61)	<.001	0.73 (0.69-0.78)	<.001
2017	0.28 (0.26-0.29)	<.001	0.21 (0.20-0.22)	<.001	0.37 (0.34-0.39)	<.001
2018	0.44 (0.42-0.47)	<.001	0.34 (0.32-0.36)	<.001	0.51 (0.48-0.54)	<.001
2019	0.52 (0.49-0.54)	<.001	0.42 (0.40-0.44)	<.001	0.63 (0.59-0.67)	<.001
Commercial beneficiaries						
2016	1 [Reference]	NA	1 [Reference]	NA	1 [Reference]	NA
2017	5.67 (5.41-5.95)	<.001	5.05 (4.83-5.27)	<.001	3.77 (3.60-3.96)	<.001
2018	7.12 (6.80-7.47)	<.001	4.90 (4.69-5.13)	<.001	3.53 (3.36-3.70)	<.001
2019	11.44 (10.92-11.98)	<.001	7.87 (7.53-8.23)	<.001	3.89 (3.70-4.09)	<.001
Medicare Advantage beneficiaries						
2016	1 [Reference]	NA	1 [Reference]	NA	1 [Reference]	NA
2017	2.25 (2.08-2.43)	<.001	1.86 (1.72-2.02)	<.001	1.88 (1.76-2.01)	<.001
2018	4.54 (4.21-4.89)	<.001	2.93 (2.71-3.18)	<.001	2.46 (2.3-2.64)	<.001
2019	8.46 (7.87-9.11)	<.001	5.83 (5.39-6.30)	<.001	3.33 (3.1-3.57)	<.001
Age, y	0.92 (0.92-0.93)	<.001	0.94 (0.94-0.95)	<.001	0.95 (0.95-0.96)	<.001
Sex						
Male	1 [Reference]	NA	1 [Reference]	NA	1 [Reference]	NA
Female	1.44 (1.4-1.48)	<.001	0.91 (0.89-0.94)	<.001	1.08 (1.05-1.11)	<.001
Race/ethnicity						
White	1 [Reference]	NA	1 [Reference]	NA	1 [Reference]	NA
Black	0.96 (0.92-0.99)	.02	0.97 (0.93-1.01)	.17	1.16 (1.12-1.21)	<.001
Hispanic	0.86 (0.83-0.90)	<.001	1.03 (0.99-1.08)	.11	1.18 (1.13-1.23)	<.001
Asian	0.49 (0.45-0.54)	<.001	0.91 (0.84-0.99)	.02	1.17 (1.08-1.27)	<.001
Unknown	0.48 (0.45-0.51)	<.001	0.50 (0.47-0.54)	<.001	0.53 (0.5-0.58)	<.001
US region						
Midwest	1 [Reference]	NA	1 [Reference]	NA	1 [Reference]	NA
Northeast	0.85 (0.81-0.90)	<.001	0.94 (0.89-0.99)	.01	1.09 (1.04-1.15)	<.001
South	1.06 (1.02-1.09)	<.001	1.11 (1.08-1.15)	<.001	1.08 (1.04-1.12)	<.001
West	0.94 (0.89-0.98)	.01	0.98 (0.93-1.03)	.35	0.94 (0.89-0.99)	.02
Annual household income, $						
<40 000	1 [Reference]	NA	1 [Reference]	NA	1 [Reference]	NA
40 000-74 999	0.97 (0.94-1.01)	.11	0.99 (0.95-1.02)	.42	0.95 (0.91-0.98)	.005
75 000-124 999	1.04 (1.00-1.08)	.04	1.01 (0.97-1.05)	.59	0.95 (0.92-0.99)	.02
125 000-199 999	1.11 (1.06-1.17)	<.001	1.05 (1.00-1.11)	.04	0.99 (0.94-1.05)	.78
≥200 000	1.23 (1.15-1.32)	<.001	1.16 (1.09-1.24)	<.001	1.06 (0.98-1.14)	.15
Unknown	0.53 (0.49-0.56)	<.001	0.52 (0.48-0.55)	<.001	0.65 (0.61-0.7)	<.001
Baseline drugs						
SGLT2i	1.97 (1.90-2.05)	<.001	NA	NA	1.22 (1.17-1.29)	<.001
GLP-1RA	NA	NA	2.04 (1.96-2.12)	<.001	0.50 (0.47-0.53)	<.001
DPP-4i	1.76 (1.70-1.82)	<.001	2.04 (1.97-2.11)	<.001	NA	NA
Metformin	1.12 (1.09-1.16)	<.001	1.20 (1.16-1.24)	<.001	1.01 (0.97-1.05)	.69
Sulfonylurea	1.44 (1.40-1.48)	<.001	1.41 (1.37-1.45)	<.001	1.53 (1.49-1.59)	<.001
Thiazolidinediones	1.29 (1.23-1.36)	<.001	1.14 (1.08-1.20)	<.001	0.90 (0.84-0.96)	.001
Basal insulin	1.85 (1.79-1.92)	<.001	1.10 (1.06-1.14)	<.001	0.78 (0.75-0.82)	<.001
Bolus insulin	1.33 (1.28-1.39)	<.001	0.97 (0.92-1.02)	.28	0.63 (0.59-0.68)	<.001
Other	1.35 (1.07-1.70)	.01	1.19 (0.92-1.53)	.18	1.04 (0.78-1.39)	.77
None	0.63 (0.60-0.68)	<.001	0.61 (0.57-0.65)	<.001	0.53 (0.50-0.57)	<.001
Treatment type						
Second-line	1 [Reference]	NA	1 [Reference]	NA	1 [Reference]	NA
First-line	0.76 (0.71-0.82)	<.001	0.51 (0.48-0.55)	<.001	0.78 (0.74-0.83)	<.001
No. of diabetes complications						
0	1 [Reference]	NA	1 [Reference]	NA	1 [Reference]	NA
1	1.15 (1.11-1.20)	<.001	1.20 (1.15-1.25)	<.001	1.06 (1.01-1.10)	.01
2	1.22 (1.15-1.31)	<.001	1.34 (1.25-1.43)	<.001	1.09 (1.02-1.17)	.02
3	1.23 (1.12-1.36)	<.001	1.41 (1.27-1.56)	<.001	1.05 (0.95-1.17)	.35
≥4	1.29 (1.12-1.48)	<.001	1.45 (1.25-1.69)	<.001	1.16 (1.00-1.36)	.06
Comorbidities						
Myocardial infarction	0.96 (0.90-1.03)	.23	1.18 (1.11-1.26)	<.001	1.07 (1.00-1.15)	.05
Cerebrovascular disease	0.92 (0.87-0.98)	.01	0.93 (0.88-0.99)	.02	1.10 (1.04-1.16)	.002
Heart failure	1.04 (0.99-1.10)	.13	0.95 (0.90-1.01)	.07	1.11 (1.05-1.17)	<.001
Nephropathy	1.17 (1.11-1.22)	<.001	0.76 (0.73-0.80)	<.001	1.28 (1.22-1.35)	<.001
Retinopathy	0.96 (0.92-1.01)	.11	0.93 (0.88-0.98)	.00	0.98 (0.93-1.04)	.48
Neuropathy	1.23 (1.18-1.28)	<.001	1.01 (0.96-1.06)	.72	1.09 (1.04-1.15)	<.001
PVD	1.03 (0.97-1.08)	.34	0.97 (0.92-1.03)	.29	1.07 (1.01-1.14)	.03
Dementia	0.94 (0.81-1.09)	.41	0.76 (0.63-0.91)	.003	1.43 (1.24-1.63)	<.001
COPD	1.10 (1.05-1.14)	<.001	1.06 (1.02-1.11)	.004	1.13 (1.08-1.18)	<.001
Cancer	0.92 (0.87-0.97)	<.001	0.91 (0.87-0.96)	<.001	1.02 (0.97-1.08)	.50
Cirrhosis	0.83 (0.74-0.93)	<.001	0.87 (0.77-0.99)	.03	1.10 (0.98-1.23)	.11
Severe hyperglycemia	0.68 (0.57-0.82)	<.001	0.71 (0.56-0.89)	.003	1.10 (0.91-1.33)	.32
Severe hypoglycemia	0.82 (0.69-0.96)	.01	0.82 (0.67-1.01)	.06	1.25 (1.07-1.47)	.01
Prescriber specialty						
Endocrinology	1 [Reference]	NA	1 [Reference]	NA	1 [Reference]	NA
Family medicine	0.34 (0.32-0.35)	<.001	0.40 (0.38-0.42)	<.001	0.77 (0.72-0.81)	<.001
Internal medicine	0.35 (0.33-0.36)	<.001	0.41 (0.39-0.43)	<.001	0.80 (0.75-0.85)	<.001
Cardiology	0.21 (0.17-0.27)	<.001	0.86 (0.75-0.98)	.02	0.68 (0.56-0.82)	<.001
Other	0.48 (0.46-0.50)	<.001	0.49 (0.46-0.52)	<.001	0.91 (0.85-0.97)	.004
Unknown	0.55 (0.52-0.57)	<.001	0.60 (0.57-0.63)	<.001	1.09 (1.02-1.17)	.01

Abbreviations: COPD, chronic obstructive pulmonary disease; DPP-4i, dipeptidyl peptidase-4 inhibitors; GLP-1RA, glucagonlike peptide-1 receptor agonists; NA, not applicable; PVD, peripheral vascular disease; SGLT2i, sodium-glucose cotransporte-2 inhibitors.

^a^ Multivariate analysis of health plan, temporal, demographic, and clinical factors associated with initiation of GLP-1RA, SGLT2i, and DPP-4i use among adults with Medicare Advantage and commercial health plans.

**Table 3.** Adjusted Proportions of Initiation of Medication to Lower Glucose Levels by Insurance Coverage, 2016 to 2019

Medication	Study year, estimated rate, %							
	Commercial health insurance				Medicare Advantage insurance			
	2016	2017	2018	2019	2016	2017	2018	2019
**All patients**								
GLP-1RA	2.14	11.04	13.49	20.02	1.50	3.32	6.48	11.44
SGLT2i	2.74	12.44	12.14	18.15	1.57	2.89	4.47	8.51
DPP-4i	3.30	11.40	10.73	11.71	2.44	4.49	5.80	7.68
**Patients with history of myocardial infarction**								
GLP-1RA	2.06	10.65	13.02	19.38	1.44	3.19	6.23	11.03
SGLT2i	3.19	14.24	13.90	20.59	1.83	3.36	5.19	9.81
DPP-4i	3.52	12.11	11.41	12.44	2.61	4.79	6.18	8.18
**Patients with cerebrovascular disease**								
GLP-1RA	2.00	10.35	12.66	18.88	1.40	3.09	6.05	10.72
SGLT2i	2.58	11.77	11.48	17.23	1.48	2.71	4.21	8.03
DPP-4i	3.58	12.28	11.57	12.62	2.65	4.87	6.28	8.30
**Patients with heart failure**								
GLP-1RA	2.22	11.40	13.92	20.60	1.56	3.44	6.70	11.81
SGLT2i	2.62	11.94	11.65	17.47	1.50	2.76	4.28	8.16
DPP-4i	3.60	12.36	11.65	12.70	2.67	4.90	6.32	8.36
**Patients with nephropathy**								
GLP-1RA	2.43	12.37	15.06	22.16	1.71	3.76	7.30	12.81
SGLT2i	2.20	10.20	9.94	15.06	1.26	2.32	3.61	6.92
DPP-4i	4.03	13.67	12.89	14.04	2.99	5.47	7.04	9.29

Abbreviations: DPP-4i, dipeptidyl peptidase-4 inhibitor; GLP-1RA, glucagonlike peptide-1 receptor agonist; SGLT2i, sodium-glucose cotransporter-2 inhibitor.

In addition to differences in initiation of medication by health plan, we found that older patients were less likely to start treatment with each of the examined medications than were younger patients, with ORs ranging from 0.92 (95% CI, 0.92-0.93) for GLP-1RA to 0.95 (95% CI, 0.95-0.96) for DPP-4i for each additional year of age. Women were more likely than men to start GLP-1RA (OR, 1.44; 95% CI, 1.40-1.48) and DPP-4i (OR, 1.08; 95% CI, 1.05-1.11) treatment but less likely to start SGLT2i treatment (OR, 0.91; 95% CI, 0.89-0.94). Compared with White patients, all non-White patients were less likely to start GLP-1RA treatment (OR for Black patients, 0.96 [95% CI, 0.92-0.99]; OR for Hispanic patients, 0.86 [95% CI, 0.83-0.90]; OR for Asian patients, 0.49 [95% CI, 0.45-0.54]), and Asian patients were also less likely to start SGLT2i treatment (OR, 0.91; 95% CI, 0.84-0.99). In contrast, Black (OR, 1.16; 95% CI, 1.12-1.21), Hispanic (OR, 1.18; 95% CI, 1.13-1.23), and Asian (OR, 1.17; 95% CI, 1.08-1.27) patients were all more likely than White patients to start DPP-4i. Patients with annual household incomes of $200 000 or higher were more likely to start GLP-1RA (OR, 1.23; 95% CI, 1.15-1.32) and SGLT2i (OR, 1.16; 95% CI, 1.09-1.24) treatment than were patients with lower household incomes lower than $40 000 (Table 2).

**Figure 2.**
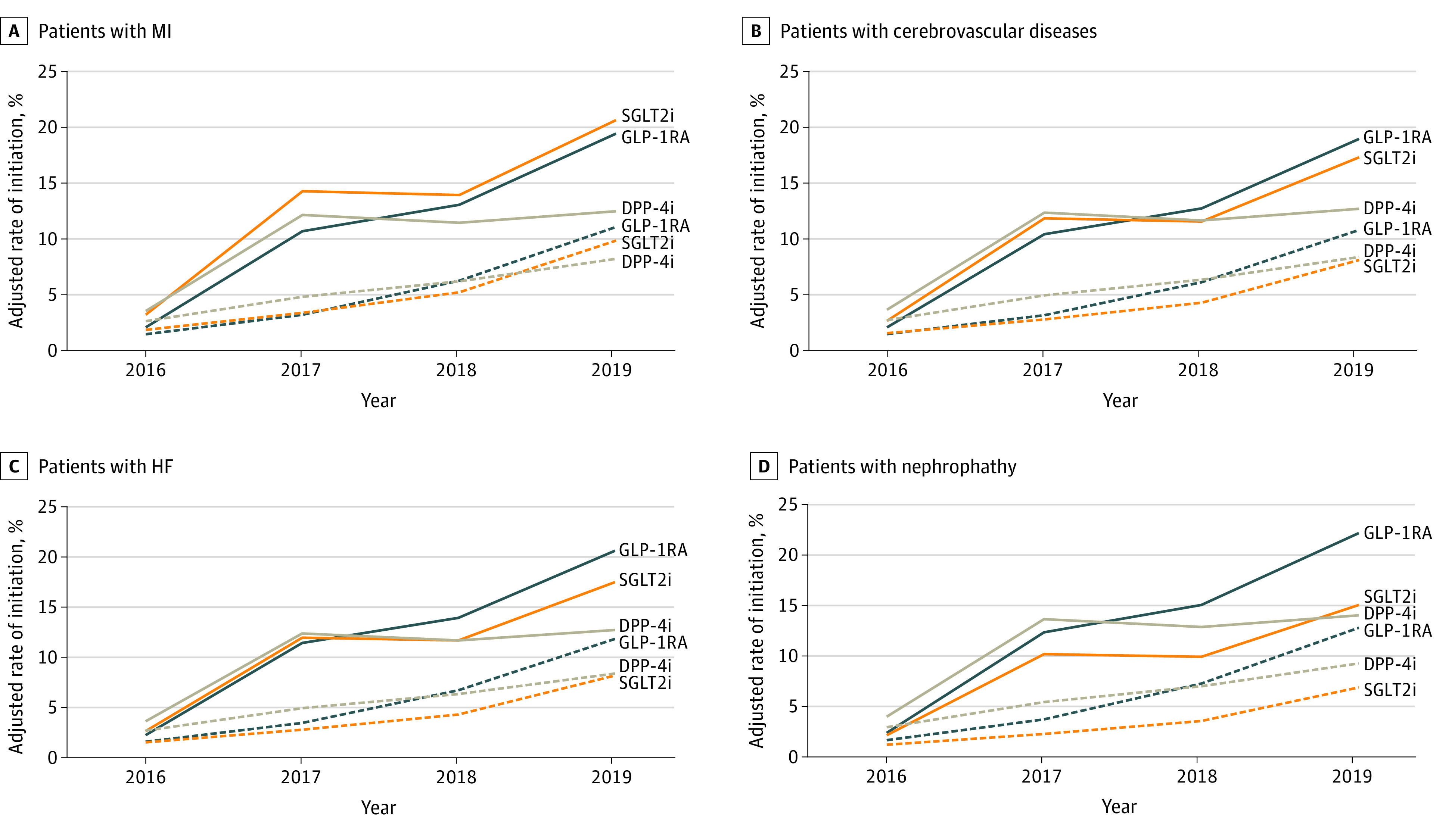
Trends in Use of Medication to Lower Glucose Levels by Older Adults With Comorbidities by Health Plan, 2016 to 2019 Solid lines represent commercial insurance coverage; dashed lines, Medicare Advantage coverage. DPP-4i indicates dipeptidyl peptidase-4 inhibitor; GLP-1RA, glucagonlike peptide-1 receptor agonist; HF, heart failure; MI, myocardial infarction; and SGLT2i, sodium-glucose cotransporter-2 inhibitor.

We also examined rates of initiation of GLP-1RA, SGLT2i, and DPP-4i treatment in subgroups of patients with comorbid health conditions that may call for preferential use of these medication classes. Patients with nephropathy, who would benefit from GLP-1RA and SGLT2i therapy, were more likely to start GLP-1RA treatment than were patients without nephropathy (OR, 1.17; 95% CI, 1.11-1.22) but were less likely to start a SGLT2i treatment (OR, 0.76; 95% CI, 0.73-0.80) (Table 2). Patients with a history of myocardial infarction, who would benefit from GLP-1RA therapy, were equally likely to start GLP-1RA treatment as were those without such history (OR, 0.96; 95% CI, 0.90-1.03). Patients with cerebrovascular disease, who would also benefit from GLP-1RA therapy, were less likely to start it (OR, 0.92; 95% CI, 0.87-0.98). Patients with heart failure, who would benefit from SGLT2i therapy and may be harmed by DPP-4i therapy, were equally likely to start a SGLT2i (OR, 0.95; 95% CI, 0.90-1.10) but were more likely to start a DPP-4i (OR, 1.11; 95% CI, 1.05-1.17) than patients without heart failure. Temporal trends in the adjusted initiation rates of medication use by patients with these 4 comorbidities paralleled that of the overall population (Figure 2), with persistent differences in initiation between Medicare Advantage and commercial insurance enrollees.

## Discussion

Contemporary approaches to managing type 2 diabetes take into consideration not only the need to control hyperglycemia but also the effects that chosen therapies may have on outcomes that matter to people with diabetes, such as cardiovascular events, kidney disease progression, and death.^3,4,5,6^ With 2 classes of medications—GLP-1RA and SGLT2i—shown to improve cardiovascular, kidney, and mortality outcomes among patients with type 2 diabetes, it was imperative to understand whether nonclinical factors, such as health insurance type, are associated with differences in medication use patterns. In this analysis of claims data for 382 574 older adults, we found that Medicare Advantage beneficiaries were significantly less likely to start GLP1-RA, SGLT2i, and DPP-4i treatment than were patients with commercial health insurance plans despite similar formulary designs.^31^

This is the first study, to our knowledge, to demonstrate the magnitudes of difference in the use GLP-1RA, SGLT2i, and DPP-4i between similarly aged enrollees in Medicare Advantage and commercial health insurance plans. These medications are not new and have a robust evidence base for their efficacy and safety, yet their use continues to lag among Medicare Advantage beneficiaries, including those who are most likely to benefit from them. Although we do not suggest an optimal rate of GLP-1RA, SGLT2i, or DPP-4i use, the gaps in their initiation among patients with preexisting high-risk conditions, such as myocardial infarction, cerebrovascular disease, heart failure, and nephropathy, suggest that an opportunity exists to improve diabetes management and health outcomes among Medicare Advantage beneficiaries.

Our findings also revealed racial/ethnic disparities in GLP-1RA use. Compared with White patients, Black, Hispanic, and Asian patients were less likely to start GLP-1RA treatment. Although there was no association between Black or Hispanic race/ethnicity and initiation of SGLT2i treatment after all other demographic and clinical factors were accounted for, Asian patients remained less likely to start SGLT2i treatment as well. Low adoption rates of new medications by Black patients have been documented previously,^23,33,34^ and these low rates may be associated with increased disparities in diabetes health outcomes.^35^ However, rates of initiation of DPP-4i treatment were higher among racial/ethnic minority groups than among White patients. Dipeptidyl peptidase-4 inhibitors are not as effective at lowering hemoglobin A_1c_ levels as either GLP-1RA or SGLT2i, nor do they have the same beneficial cardiovascular or kidney effects.^3^ Why DPP-4i treatment is started more frequently by patients who are more likely to benefit from alternative medication classes and why initiation rates are higher among racial/ethnic minorities is unknown. Until recently, some guidelines^4^ recommended DPP-4i as second-line therapy preferred to GLP-1RA or SGLT2i owing to the benign adverse effect profile and ease of administration of the drug class (ie, oral formulation), but with emergence of robust efficacy data for GLP-1RA and SGLT2i, these 2 classes are now increasingly preferred to DPP-4i medications.^3,7^ A more nuanced understanding of factors associated with medication preferences, including qualitative exploration of clinicians’ and patients’ attitudes and beliefs about the selection of medication to lower glucose levels, therefore appears to be needed.

We hypothesize that some of the differences in the use of GLP-1RA, SGLT2i, and DPP-4i medications may be associated with affordability because all are costly brand-name medications. Patients with Medicare Advantage plans are ineligible for manufacturer savings cards and, at some point during the year, may face a “donut hole” with limited or no prescription drug coverage, both of which may make out-of-pocket cost-sharing expenses more unaffordable for them than for commercial insurance enrollees. A total of 40.2% of Medicare Advantage beneficiaries in the present cohort had an annual household income below $40 000 compared with 18.6% of commercial beneficiaries, and lower incomes were significantly associated with decreased the odds of initiating GLP-1RA and SGLT2i treatment. We did not observe a consistent association between income and initiation of DPP-4i treatment, however, potentially because DPP-4i medications are less costly than GLP-1RA and SGLT2i medications even if they are more expensive than generic alternatives.^3^ Lower income is associated with worse glycemic control and diabetes-related health outcomes compared with higher incomes,^36,37,38,39,40,41,42^ and financial barriers to optimal pharmacotherapy may contribute to these differences.

### Limitations

This study has limitations. It relied on data from commercial insurance and Medicare Advantage plans offered by a single, large, national health insurance company, which allowed for direct comparisons between commercial insurance and Medicare Advantage plans, but results may not generalize to other private and public health plans. Our analyses focused on initiation of medication rather than adherence and persistence, and as such, our findings likely underestimate the association of cost-related barriers with evidence-based medication use. We were unable to capture medications obtained as samples or through manufacturer financial assistance programs, which may underestimate the use of costly medications, particularly among individuals in lower-income households. We were also unable to identify the clinical contexts (including hemoglobin A_1c_ level) and shared decision-making conversations between patients and their clinicians that may have affected medication choice and differed between commercial insurance plan and Medicare Advantage enrollees. Thus, these findings suggest the need for better understanding of factors contributing to treatment decisions and efforts to improve affordability and equity in the use of glucose-lowering therapies by all patients with diabetes.

## Conclusions

Our study identified gaps in the use of effective, safe, and guideline-recommended medications to lower glucose levels among patients with Medicare Advantage health insurance, particularly among patients who may benefit from preferential use of these medications, such as those with underlying cardiovascular or kidney disease. The effect of the observed disparities may be unknown until the long-term benefits of these agents are more definitively established. Initiation of GLP-1RA and SGLT2i treatment was also reduced among individuals in lower-income households, potentially contributing to poor health outcomes in this population. The findings suggest that innovative solutions are needed to address logistical and financial barriers to evidence-based diabetes management, including simplifying coverage requirements, reducing cost-sharing responsibilities, and creating transparent and straightforward avenues for all patients to obtain and afford the medications they need to optimally manage their disease. Such efforts may help improve access to new diabetes therapeutics, reduce disparities, and improve the health outcomes for patients living with diabetes.

## Notes

Supplement. **eTable 1.** Classification Scheme for Medications to Lower Glucose Levels **eTable 2.** Code Sets for Included Comorbidities **eTable 3.** Characteristics of Older Adults With Type 2 Diabetes Initiating GLP-1 Receptor Agonist Therapy, 2016 to 2019 **eTable 4.** Characteristics of Older Adults With Type 2 Diabetes Initiating SGLT2 Inhibitor Therapy, 2016 to 2019 **eTable 5.** Characteristics of Older Adults With Type 2 Diabetes Initiating DPP-4 Inhibitor Therapy, 2016-2019 **eFigure.** Age Distribution of Medicare Advantage and Commercial Health Plan Beneficiaries With Type 2 Diabetes Included in OptumLabs Data Warehouse From 2016 to 2019
