# Supplementary material for: Comparison of Diabetes Medications Used by Adults With Commercial Insurance vs Medicare Advantage, 2016 to 2019
Source: JAMA Netw Open. 2021 Feb 1;4(2):e2035792. doi: 10.1001/jamanetworkopen.2020.35792 (PMC7851726; doi:10.1001/jamanetworkopen.2020.35792)
Supplement: Supplement. — eTable 1. Classification Scheme for Medications to Lower Glucose Levels eTable 2. Code Sets for Included Comorbidities eTable 3. Characteristics of Older Adults With Type 2 Diabetes Initiating GLP-1 Receptor Agonist Therapy, 2016 to 2019 eTable 4. Characteristics of Older Adults With Type 2 Diabetes Initiating SGLT2 Inhibitor Therapy, 2016 to 2019 eTable 5. Characteristics of Older Adults With Type 2 Diabetes Initiating DPP-4 Inhibitor Therapy, 2016-2019 eFigure. Age Distribution of Medicare Advantage and Commercial Health Plan Beneficiaries With Type 2 Diabetes Included in OptumLabs Data Warehouse From 2016 to 2019 [file jamanetwopen-e2035792-s001.pdf]

Supplementary Online Content

McCoy RG, Van Houten HK, Deng Y, et al. Comparison of diabetes medications used by adults with commercial insurance vs Medicare Advantage, 2016 to 2019. *JAMA Netw Open*. 2021;4(2):e2035792. doi:10.1001/jamanetworkopen.2020.35792

eTable 1. Classification Scheme for Medications to Lower Glucose Levels
eTable 2. Code Sets for Included Comorbidities
eTable 3. Characteristics of Older Adults With Type 2 Diabetes Initiating GLP-1 Receptor Agonist Therapy, 2016 to 2019
eTable 4. Characteristics of Older Adults With Type 2 Diabetes Initiating SGLT2 Inhibitor Therapy, 2016 to 2019
eTable 5. Characteristics of Older Adults With Type 2 Diabetes Initiating DPP-4 Inhibitor Therapy, 2016-2019
eFigure. Age Distribution of Medicare Advantage and Commercial Health Plan Beneficiaries With Type 2 Diabetes Included in OptumLabs Data Warehouse From 2016 to 2019

This supplementary material has been provided by the authors to give readers additional information about their work.

©2021 MCoy R et al. *JAMA Network Open*.

eTable 1. Classification Scheme for Medications to Lower Glucose Levels
For GLP-1 receptor agonists, SGLT2 inhibitors, and DPP-4 inhibitors, date of first FDA approval are presented parenthetically.

	Medication class	Included agents
	GLP-1 receptor agonists	Exenatide (04/2005)<br>Liraglutide (01/2010)<br>Albiglutide (05/2014)<br>Dulaglutide (09/2014)<br>Lixisenatide (07/2016)<br>Semaglutide (12/2017)
	SGLT-2 inhibitors	Canagliflozin (03/2013)<br>Empagliflozin (08/2014)<br>Dapagliflozin (01/2014)<br>Ertugliflozin (12/2017)
	DPP-4 inhibitors	Sitagliptin (10/2006)<br>Saxagliptin (07/2009)<br>Linagliptin (05/2011)<br>Alogliptin (01/2013)
**Insulin**	Insulin: Basal	NPH/isophane<br>Determir<br>Glargine<br>Degludec
	Insulin: Bolus	Regular<br>Aspart<br>Lispro<br>Glulisine<br>Inhaled powder insulin
**Other Medications**	Biguanides	Metformin
	Sulfonylureas	Acetohexamide<br>Chlorpropamide<br>Glimepiride<br>Glipizide<br>Glyburide<br>Tolazamide<br>Tolbutamide
	Glitazones	Pioglitazone<br>Rosiglitazone<br>Troglitazone
	Glinides	Nateglinide<br>Repaglinide
	Amylin analogs	Pramlintide
	α-Glucosidase inhibitors	Acarbose<br>Miglitol

©2021 MCoy R et al. *JAMA Network Open*.

eTable 2. Code Sets for Included Comorbidities

Comorbidity	ICD-9 Codes	ICD-10 codes
Myocardial infarction	410.xx, 412	I21.xx, I22.x, I25.2
Cerebrovascular disease	430, 431, 432.x, 433.xx, 434.xx 435.x, 436, 437.x, 438.xx, V12.54	G45.0, G45.1, G45.2, G45.8, G45.9, G46.x, I60.xx, I61.x (except I61.0), I62.xx, I63.xxx, I65.xx, I66.xx, I67.8x (except I67.83, I67.84), I67.9, I69.xxx, Z86.73
Heart failure	398.91, 402.01, 402.11, 402.91, 404.01, 404.03, 404.11, 404.13, 404.91, 404.93, 428.xx	I09.81, I11.0, I13.0, I13.2, I50.xx
Nephropathy	593.9, 586, 250.4x, 249.4x, 580.x, 581.x, 582.x, 583.x, 585.x	E08.21, E08.22, E08.29, E09.21, E09.22, E09.29, E10.21, E10.22, E10.29, E11.21, E11.22, E11.29, E13.21, E13.22, E13.29, N19, N00.x, N03.x, N04.x, N05.x, N18.x
Retinopathy	362.01, 362.03, 362.04, 362.05, 362.06, 362.07, 362.53, 362.81, 362.82, 362.83, 362.02, 379.23, 250.5x, 249.5x, 362.1x, 361.x, 369.x	H35.9, E08.3x, E09.3x, E10.3x, E11.3x, E13.3x, H35.0x, H35.35x, H35.6x, H35.8x, H33.x, H54.x, H43.1x
Neuropathy	357.2, 337.1, 356.9, 358.1, 458.0, 536.3, 564.5, 596.54, 713.5, 951.0, 951.1, 951.3, 250.6x, 249.6x, 337.0x, 354.x, 355.x	G90.09, G90.8, G90.9, G99.0, G60.9, G73.3, G90.01, I95.1, K31.84, K59.1, N31.9, E08.4x, E09.4x, E10.4x, E11.4x, E13.4x, G56.x, G57.x, H49.x, M14.6x, S04.x
Peripheral vascular disease	442.3, 440.21, 443.81, 443.9, 892.1, 040.0, 444.22, 785.4, 250.7x, 249.7, 707.1x	E08.51, E09.51, E10.51, E11.51, E13.51, E08.59, E09.59, E10.59, E11.59, E13.59, E08.621, E09.621, E10.621, E11.621, E13.621, I72.4, I73.89, I73.9, A48.0, I74.3, I96, E08.52, E09.52, E10.52, E11.52, E13.52, I70.21x, S91.3x, L97.x
Dementia	046.1x, 290.1x, 290.2x, 290.3, 290.4x, 291.2, 292.82, 294.1x, 294.2x, 331.0, 331.1x, 331.2, 331.6, 331.82, 331.89	A81.0x, F01.5x, F02.8x, F03.9x, F10.27, F10.97, F13.27, F13.97, F18.97, F19.17, F19.27, F19.97, G30.x, G31.0x, G31.1, G31.83, G31.85
Chronic obstructive pulmonary disease	490, 491.xx, 492.x, 494.x, 496	J40, J41.x, J42, J43.x, J44.x, J47.x
Cirrhosis	571.2, 571.5, 571.6	K70.3x, K74.3, K74.4, K74.5, K74.6x
Cancer (except non-melanoma skin cancer)	14x.xx, 15x.xx, 160.x-165.x, 170.x-172.x, 174.x-176.x, 179-189.x, 190.x-195.x, 199.xx-208.xx (except 203.x1, 204.x1, 205.x1, 206.x1, 207.x1, 208.x1)	C00.x-C14.x, C15.x-C26.x, C3x.xx, C40.xx-C41.x, C43.x, C4A.xx, C45.x-C49.xx, C50.xxx, C51.x-C58, C60.x-C63.x, C64.x-C68.x, C69.xx-C72.x, C73-C76.x, C7A.xx, C80.xx-C96.x (except C90.x1, C91.x1, C92.x1, C93.x1, C94.x1, C95.x1)
Hypoglycemia	251.0, 251.1, 251.2, 270.3, 962.3, 250.8x (for 250.8x: if no concurrent 259.8, 272.7, 681.xx, 682.xx, 686.9x, 707.1x-707.2x, 707.8, 707.9, 709.3, 730.0x-730.2x, 731.8)	E10.641, E10.649, E11.641, E11.649, E13.641, E13.649, E16.0, E16.1, E16.2, T38.3X1A, T38.3X1D, T38.3X1S, T38.3X2A, T38.3X2D, T38.3X2S, T38.3X3A, T38.3X3D, T38.3X3S, T38.3X4A, T383X4D, T38.3X4S, T38.3X5A, T383X5D, T38.3X5S
Hyperglycemia	250.10, 250.11, 250.12, 250.13, 250.20, 250.21, 250.22, 250.23	E10.10, E10.11, E11.00, E11.01, E11.10, E11.11, E13.00, E13.01, E13.10, E13.11

eTable 3. Characteristics of Older Adults with Type 2 Diabetes Initiating GLP-1 Receptor Agonist Therapy, 2016 to 2019

Univariate comparisons of health plan, temporal, demographic, and clinical factors associated with GLP-1 receptor agonist initiation among adults with Medicare Advantage and commercial health plans.

	GLP1 Non-initiators (N=329,541)	GLP1 Initiators (N=29,844)	OR (95%CI)	p value
**Health plan, N (%)**				
Commercial	181762 (55.2%)	19452 (65.2%)	1 [Reference]	NA
Medicare Advantage	147779 (44.8%)	10392 (34.8%)	0.66 (0.64, 0.67)	<0.001
**Index year, N (%)**				
2016	140208 (42.5%)	4778 (16.0%)	1 [Reference]	NA
2017	70977 (21.5%)	6668 (22.3%)	2.76 (2.65, 2.86)	<0.001
2018	61045 (18.5%)	8207 (27.5%)	3.95 (3.80, 4.09)	<0.001
2019	57311 (17.4%)	10191 (34.1%)	5.22 (5.04, 5.41)	<0.001
**Age, mean (SD)**	62.4 (2.7)	61.8 (2.5)	0.92 (0.92, 0.92)	<0.001
**Gender, N (%)**				
Male	152220 (46.2%)	15329 (51.4%)	1 [Reference]	NA
Female	177321 (53.8%)	14515 (48.6%)	1.23 (1.20, 1.26)	<0.001
**Race/ethnicity, N (%)**				
White	192816 (58.5%)	19218 (64.4%)	1 [Reference]	NA
Black	53132 (16.1%)	4671 (15.7%)	0.88 (0.85, 0.91)	<0.001
Hispanic	40041 (12.2%)	3397 (11.4%)	0.85 (0.82, 0.88)	<0.001
Asian	10195 (3.1%)	494 (1.7%)	0.49 (0.44, 0.53)	<0.001
Unknown	33357 (10.1%)	2064 (6.9%)	0.62 (0.59, 0.65)	<0.001
**U.S. Region, N (%)**				
Midwest	81253 (24.7%)	7494 (25.1%)	1 [Reference]	NA
Northeast	34174 (10.4%)	2584 (8.7%)	0.82 (0.78, 0.86)	<0.001
South	176486 (53.6%)	16552 (55.5%)	1.02 (0.99, 1.05)	0.25
West	37628 (11.4%)	3214 (10.8%)	0.93 (0.89, 0.97)	<0.001
**Annual household income, N (%)**				
<$40,000	93961 (28.5%)	8324 (27.9%)	1 [Reference]	NA
$40,000 - $74,999	79682 (24.2%)	7043 (23.6%)	1.00 (0.97, 1.03)	0.89
$75,000 - $124,999	75062 (22.8%)	7432 (24.9%)	1.12 (1.08, 1.16)	<0.001
$125,000 - $199,999	27795 (8.4%)	3098 (10.4%)	1.26 (1.21, 1.31)	<0.001
≥200,000	12243 (3.7%)	1515 (5.1%)	1.40 (1.32, 1.48)	<0.001
Unknown	40798 (12.4%)	2432 (8.1%)	0.67 (0.64, 0.71)	<0.001
**Baseline medications, N (%)**				
GLP-1RA	9808 (3.0%)	-	-	-

©2021 MCoy R et al. *JAMA Network Open*.

SGLT2i	19358 (5.9%)	4992 (16.7%)	3.22 (3.11, 3.33)	<0.001
DPP-4i	33704 (10.2%)	6116 (20.5%)	2.26 (2.20, 2.33)	<0.001
Metformin	174130 (52.8%)	18414 (61.7%)	1.44 (1.40, 1.47)	<0.001
Sulfonylurea	71196 (21.6%)	9164 (30.7%)	1.61 (1.57, 1.65)	<0.001
Thiazolidinedione	15084 (4.6%)	2053 (6.9%)	1.54 (1.47, 1.62)	<0.001
Basal insulin	48758 (14.8%)	8638 (28.9%)	2.35 (2.28, 2.41)	<0.001
Bolus insulin	25477 (7.7%)	4562 (15.3%)	2.15 (2.08, 2.23)	<0.001
Other	627 (0.2%)	100 (0.3%)	1.76 (1.43, 2.18)	<0.001
None	99768 (30.3%)	4374 (14.7%)	0.40 (0.38, 0.41)	<0.001
**Treatment type, N (%)**				
First-line	267851 (81.3%)	26965 (90.4%)	0.46 (0.45, 0.48)	<0.001
Second-line	61690 (18.7%)	2879 (9.6%)	1 [Reference]	NA
**Count of diabetes complications, N (%)**				
0	149891 (45.5%)	11135 (37.3%)	1 [Reference]	NA
1	94069 (28.5%)	8910 (29.9%)	1.28 (1.24, 1.31)	<0.001
2	47392 (14.4%)	5201 (17.4%)	1.48 (1.43, 1.53)	<0.001
3	23428 (7.1%)	2732 (9.2%)	1.57 (1.50, 1.64)	<0.001
≥4	14761 (4.5%)	1866 (6.3%)	1.70 (1.62, 1.79)	<0.001
**Comorbidities, N (%)**				
Myocardial infarction	12482 (3.8%)	1193 (4.0%)	1.06 (1.00, 1.12)	0.07
Cerebrovascular disease	28376 (8.6%)	2525 (8.5%)	0.98 (0.94, 1.02)	0.38
Heart failure	27104 (8.2%)	2715 (9.1%)	1.12 (1.07, 1.16)	<0.001
Nephropathy	51064 (15.5%)	6120 (20.5%)	1.41 (1.37, 1.45)	<0.001
Retinopathy	40421 (12.3%)	4539 (15.2%)	1.28 (1.24, 1.33)	<0.001
Neuropathy	76973 (23.4%)	9473 (31.7%)	1.53 (1.49, 1.57)	<0.001
Peripheral vascular disease	39789 (12.1%)	4401 (14.7%)	1.26 (1.22, 1.30)	<0.001
Dementia	3151 (1.0%)	213 (0.7%)	0.75 (0.65, 0.86)	<0.001
COPD	45011 (13.7%)	4174 (14.0%)	1.03 (0.99, 1.06)	0.11
Cancer	23442 (7.1%)	1990 (6.7%)	0.93 (0.89, 0.98)	0.004
Cirrhosis	4833 (1.5%)	405 (1.4%)	0.92 (0.84, 1.02)	0.13
Severe hyperglycemia	1669 (0.5%)	144 (0.5%)	0.95 (0.80, 1.31)	0.58
Severe hypoglycemia	2036 (0.6%)	184 (0.6%)	1.00 (0.86, 1.16)	1.00
**Prescriber specialty, N (%)**				
Endocrinology	18677 (5.7%)	5096 (17.1%)	1 [Reference]	NA
Family medicine	127877 (38.8%)	8914 (29.9%)	0.26 (0.25, 0.27)	<0.001
Internal medicine	92719 (28.1%)	6394 (21.4%)	0.25 (0.24, 0.26)	<0.001
Cardiology	2330 (0.7%)	86 (0.3%)	0.14 (0.11, 0.17)	<0.001
Other	33907 (10.3%)	4000 (13.4%)	0.43 (0.41, 0.45)	<0.001
Unknown	54031 (16.4%)	5354 (17.9%)	0.36 (0.35, 0.38)	<0.001

©2021 MCoy R et al. *JAMA Network Open*.

eTable 4. Characteristics of Older Adults with Type 2 Diabetes Initiating SGLT2 Inhibitor Therapy, 2016 to 2019

Univariate comparisons of health plan, temporal, demographic, and clinical factors associated with SGLT2 inhibitor initiation among adults with Medicare Advantage and commercial health plans.

	SGLT2 Non-initiators (N=333,749)	SGLT2 Initiators (N=27,209)	OR (95%CI)	p value
**Health plan, N (%)**				
Commercial	180810 (54.2%)	19648 (72.2%)	1 [Reference]	NA
Medicare Advantage	152939 (45.8%)	7561 (27.8%)	0.46 (0.44, 0.47)	<0.001
**Index year, N (%)**				
2016	140510 (42.1%)	5596 (20.6%)	1 [Reference]	NA
2017	72053 (21.6%)	6670 (24.5%)	2.32 (2.24, 2.41)	<0.001
2018	61992 (18.6%)	6516 (23.9%)	2.64 (2.54, 2.74)	<0.001
2019	59194 (17.7%)	8427 (31.0%)	3.57 (3.45, 3.70)	<0.001
**Age, mean (SD)**	62.4 (2.7)	61.8 (2.5)	0.92 (0.91, 0.92)	<0.001
**Gender, N (%)**				
Male	159071 (47.7%)	11526 (42.4%)	1 [Reference]	NA
Female	174678 (52.3%)	15683 (57.6%)	0.81 (0.79, 0.83)	<0.001
**Race/ethnicity, N (%)**				
White	195321 (58.5%)	17430 (64.1%)	1 [Reference]	NA
Black	54571 (16.4%)	3786 (13.9%)	0.78 (0.75, 0.81)	<0.001
Hispanic	39794 (11.9%)	3493 (12.8%)	0.98 (0.95, 1.02)	0.39
Asian	9659 (2.9%)	786 (2.9%)	0.91 (0.85, 0.98)	0.02
Unknown	34404 (10.3%)	1714 (6.3%)	0.56 (0.53, 0.58)	<0.001
**U.S. Region, N (%)**				
Midwest	82604 (24.8%)	6609 (24.3%)	1 [Reference]	NA
Northeast	34303 (10.3%)	2474 (9.1%)	0.90 (0.86, 0.95)	<0.001
South	178999 (53.6%)	14926 (54.9%)	1.04 (1.01, 1.07)	0.007
West	37843 (11.3%)	3200 (11.8%)	1.06 (1.01, 1.10)	0.01
**Annual household income, N (%)**				
<$40,000	96356 (28.9%)	6782 (24.9%)	1 [Reference]	NA
$40,000 - $74,999	80438 (24.1%)	6614 (24.3%)	1.17 (1.13, 1.21)	<0.001
$75,000 - $124,999	75299 (22.6%)	7153 (26.3%)	1.35 (1.30, 1.40)	<0.001
$125,000 - $199,999	27592 (8.3%)	3085 (11.3%)	1.59 (1.52, 1.66)	<0.001
≥200,000	12048 (3.6%)	1618 (5.9%)	1.91 (1.80, 2.02)	<0.001
Unknown	42016 (12.6%)	1957 (7.2%)	0.66 (0.63, 0.70)	<0.001
**Baseline medications, N (%)**				
GLP-1RA	19566 (5.9%)	4512 (16.6%)	3.19 (3.08, 3.31)	<0.001

©2021 MCoy R et al. *JAMA Network Open*.

SGLT2i	11134 (3.3%)	-	-	-
DPP-4i	32200 (9.6%)	5553 (20.4%)	2.40 (2.33, 2.48)	<0.001
Metformin	173723 (52.1%)	18248 (67.1%)	1.88 (1.83, 1.93)	<0.001
Sulfonylurea	70983 (21.3%)	8765 (32.2%)	1.76 (1.71, 1.81)	<0.001
Thiazolidinedione	15058 (4.5%)	1869 (6.9%)	1.56 (1.49, 1.64)	<0.001
Basal insulin	53827 (16.1%)	5476 (20.1%)	1.31 (1.27, 1.35)	<0.001
Bolus insulin	28370 (8.5%)	2589 (9.5%)	1.13 (1.09, 1.18)	<0.001
Other	637 (0.2%)	77 (0.3%)	1.49 (1.17, 1.88)	<0.001
None	100731 (30.2%)	3522 (12.9%)	0.34 (0.33, 0.36)	<0.001
**Treatment type, N (%)**				
First-line	271463 (81.3%)	25146 (92.4%)	0.36 (0.34, 0.37)	<0.001
Second-line	62286 (18.7%)	2063 (7.6%)	1 [Reference]	NA
**Count of diabetes complications, N (%)**				
0	145137 (43.5%)	11214 (41.2%)	1 [Reference]	NA
1	94448 (28.3%)	8274 (30.4%)	1.13 (1.10, 1.17)	<0.001
2	50003 (15.0%)	4399 (16.2%)	1.14 (1.10, 1.18)	<0.001
3	26014 (7.8%)	2123 (7.8%)	1.06 (1.01, 1.11)	0.03
≥4	18147 (5.4%)	1199 (4.4%)	0.86 (0.80, 0.91)	<0.001
**Comorbidities, N (%)**				
Myocardial infarction	16655 (5.0%)	1484 (5.5%)	1.10 (1.04, 1.16)	<0.001
Cerebrovascular disease	32568 (9.8%)	2266 (8.3%)	0.84 (0.80, 0.88)	<0.001
Heart failure	30209 (9.1%)	2039 (7.5%)	0.81 (0.78, 0.85)	<0.001
Nephropathy	57442 (17.2%)	4128 (15.2%)	0.86 (0.83, 0.89)	<0.001
Retinopathy	43372 (13.0%)	3662 (13.5%)	1.04 (1.01, 1.08)	0.03
Neuropathy	84582 (25.3%)	7331 (26.9%)	1.09 (1.06, 1.12)	<0.001
Peripheral vascular disease	43582 (13.1%)	3536 (13.0%)	1.00 (0.96, 1.03)	0.77
Dementia	3699 (1.1%)	134 (0.5%)	0.44 (0.37, 0.53)	<0.001
COPD	49657 (14.9%)	3412 (12.5%)	0.82 (0.79, 0.85)	<0.001
Cancer	24516 (7.3%)	1735 (6.4%)	0.86 (0.82, 0.90)	<0.001
Cirrhosis	5227 (1.6%)	316 (1.2%)	0.74 (0.66, 0.83)	<0.001
Severe hyperglycemia	1751 (0.5%)	84 (0.3%)	0.59 (0.47, 0.73)	0.58
Severe hypoglycemia	2191 (0.7%)	110 (0.4%)	0.61 (0.51, 0.74)	1.00
**Prescriber specialty, N (%)**				
Endocrinology	19804 (5.9%)	4144 (15.2%)	1 [Reference]	NA
Family medicine	128077 (38.4%)	8922 (32.8%)	0.33 (0.32, 0.35)	<0.001
Internal medicine	92976 (27.9%)	6357 (23.4%)	0.33 (0.31, 0.34)	<0.001
Cardiology	2242 (0.7%)	306 (1.1%)	0.65 (0.58, 0.74)	<0.001
Other	34390 (10.3%)	3530 (13.0%)	0.49 (0.47, 0.52)	<0.001
Unknown	56260 (16.9%)	3950 (14.5%)	0.34 (0.32, 0.35)	<0.001

eTable 5. Characteristics of Older Adults with Type 2 Diabetes Initiating DPP-4 Inhibitor Therapy, 2016-2019

Univariate comparisons of health plan, temporal, demographic, and clinical factors associated with DPP-4 inhibitor initiation among adults with Medicare Advantage and commercial health plans.

	DPP4 Non-initiators (N=328,684)	DPP4 Initiators (N=23,094)	OR (95%CI)	p value
**Health plan, N (%)**				
Commercial	184940 (56.3%)	14396 (62.3%)	1 [Reference]	NA
Medicare Advantage	143744 (43.7%)	8698 (37.7%)	0.78 (0.76, 0.80)	<0.001
**Index year, N (%)**				
2016	142247 (43.3%)	5727 (24.8%)	1 [Reference]	NA
2017	67696 (20.6%)	6197 (26.8%)	2.27 (2.19, 2.36)	<0.001
2018	60256 (18.3%)	5783 (25.0%)	2.38 (2.30, 2.48)	<0.001
2019	58485 (17.8%)	5387 (23.3%)	2.29 (2.20, 2.38)	<0.001
**Age, mean (SD)**	62.3 (2.7)	62.0 (2.5)	0.95 (0.95, 0.96)	<0.001
**Gender, N (%)**				
Male	154140 (46.9%)	10844 (47.0%)	1 [Reference]	NA
Female	174544 (53.1%)	12250 (53.0%)	1.00 (0.98, 1.03)	0.86
**Race/ethnicity, N (%)**				
White	196074 (59.7%)	13672 (59.2%)	1 [Reference]	NA
Black	51597 (15.7%)	4071 (17.6%)	1.13 (1.09, 1.17)	<0.001
Hispanic	38486 (11.7%)	3181 (13.8%)	1.19 (1.14, 1.23)	<0.001
Asian	9283 (2.8%)	751 (3.3%)	1.16 (1.08, 1.25)	<0.001
Unknown	33244 (10.1%)	1419 (6.1%)	0.61 (0.58, 0.65)	<0.001
**U.S. Region, N (%)**				
Midwest	82257 (25.0%)	5442 (23.6%)	1 [Reference]	NA
Northeast	33156 (10.1%)	2315 (10.0%)	1.06 (1.00, 1.11)	0.04
South	175226 (53.3%)	12862 (55.7%)	1.11 (1.07, 1.15)	<0.001
West	38045 (11.6%)	2475 (10.7%)	0.98 (0.94, 1.03)	0.50
**Annual household income, N (%)**				
<$40,000	92549 (28.2%)	6896 (29.9%)	1 [Reference]	NA
$40,000 - $74,999	79349 (24.1%)	5686 (24.6%)	0.96 (0.93, 1.00)	0.04
$75,000 - $124,999	75878 (23.1%)	5444 (23.6%)	0.96 (0.93, 1.00)	0.04
$125,000 - $199,999	28388 (8.6%)	2155 (9.3%)	1.02 (0.97, 1.07)	0.47
≥200,000	12555 (3.8%)	1009 (4.4%)	1.08 (1.01, 1.16)	0.03
Unknown	39965 (12.2%)	1904 (8.2%)	0.64 (0.61, 0.67)	<0.001
**Baseline medications, N (%)**				
GLP-1RA	23361 (7.1%)	1146 (5.0%)	0.68 (0.64, 0.73)	<0.001

©2021 MCoy R et al. *JAMA Network Open*.

SGLT2i	21416 (6.5%)	2108 (9.1%)	1.44 (1.38, 1.51)	<0.001
DPP-4i	18694 (5.7%)	-	-	-
Metformin	172447 (52.5%)	14151 (61.3%)	1.43 (1.40, 1.47)	<0.001
Sulfonylurea	68850 (20.9%)	7488 (32.4%)	1.81 (1.76, 1.86)	<0.001
Thiazolidinedione	15214 (4.6%)	1155 (5.0%)	1.09 (1.02, 1.15)	<0.001
Basal insulin	54510 (16.6%)	3190 (13.8%)	0.81 (0.78, 0.84)	<0.001
Bolus insulin	29180 (8.9%)	1391 (6.0%)	0.66 (0.62, 0.70)	<0.001
Other	628 (0.2%)	52 (0.2%)	1.18 (0.89, 1.57)	0.25
None	98851 (30.1%)	4627 (20.0%)	0.58 (0.56, 0.60)	<0.001
**Treatment type, N (%)**				
First-line	267385 (81.4%)	20131 (87.2%)	0.64 (0.62, 0.67)	<0.001
Second-line	61299 (18.6%)	2963 (12.8%)	1 [Reference]	NA
**Count of diabetes complications, N (%)**				
0	148152 (45.1%)	9732 (42.1%)	1 [Reference]	NA
1	94010 (28.6%)	6688 (29.0%)	1.08 (1.05, 1.12)	<0.001
2	47806 (14.5%)	3627 (15.7%)	1.16 (1.11, 1.20)	<0.001
3	23815 (7.2%)	1782 (7.7%)	1.14 (1.08, 1.20)	<0.001
≥4	14901 (4.5%)	1265 (5.5%)	1.29 (1.22, 1.37)	<0.001
**Comorbidities, N (%)**				
Myocardial infarction	12431 (3.8%)	990 (4.3%)	1.14 (1.07, 1.22)	<0.001
Cerebrovascular disease	27949 (8.5%)	2160 (9.4%)	1.11 (1.06, 1.16)	<0.001
Heart failure	26969 (8.2%)	2112 (9.1%)	1.13 (1.08, 1.18)	<0.001
Nephropathy	51079 (15.5%)	4324 (18.7%)	1.25 (1.21, 1.30)	<0.001
Retinopathy	41178 (12.5%)	2899 (12.6%)	1.00 (0.96, 1.04)	0.91
Neuropathy	78920 (24.0%)	5967 (25.8%)	1.10 (1.07, 1.14)	<0.001
Peripheral vascular disease	39799 (12.1%)	3088 (13.4%)	1.12 (1.08, 1.17)	<0.001
Dementia	2908 (0.9%)	243 (1.1%)	1.19 (1.05, 1.36)	0.009
COPD	44588 (13.6%)	3300 (14.3%)	1.06 (1.02, 1.10)	0.002
Cancer	23146 (7.0%)	1619 (7.0%)	1.00 (0.95, 1.05)	0.86
Cirrhosis	4749 (1.4%)	345 (1.5%)	1.04 (0.93, 1.16)	0.54
Severe hyperglycemia	1678 (0.5%)	123 (0.5%)	1.04 (0.87, 1.26)	0.64
Severe hypoglycemia	2060 (0.6%)	178 (0.8%)	1.23 (1.06, 1.44)	0.008
**Prescriber specialty, N (%)**				
Endocrinology	21828 (6.6%)	1688 (7.3%)	1 [Reference]	NA
Family medicine	126113 (38.4%)	8161 (35.3%)	0.84 (0.79, 0.88)	<0.001
Internal medicine	91020 (27.7%)	6053 (26.2%)	0.86 (0.81, 0.91)	<0.001
Cardiology	2217 (0.7%)	127 (0.5%)	0.74 (0.62, 0.89)	0.002
Other	34621 (10.5%)	2870 (12.4%)	1.07 (1.01, 1.14)	0.03
Unknown	52885 (16.1%)	4195 (18.2%)	1.03 (0.97, 1.09)	0.40

©2021 MCoy R et al. *JAMA Network Open*.

eFigure. Age Distribution of Medicare Advantage and Commercial Health Plan Beneficiaries With Type 2 Diabetes Included in OptumLabs Data Warehouse From 2016 to 2019
At least 1% of people in each one-year subset of both the Medicare Advantage and commercial cohorts were aged 58 through 66 years old.

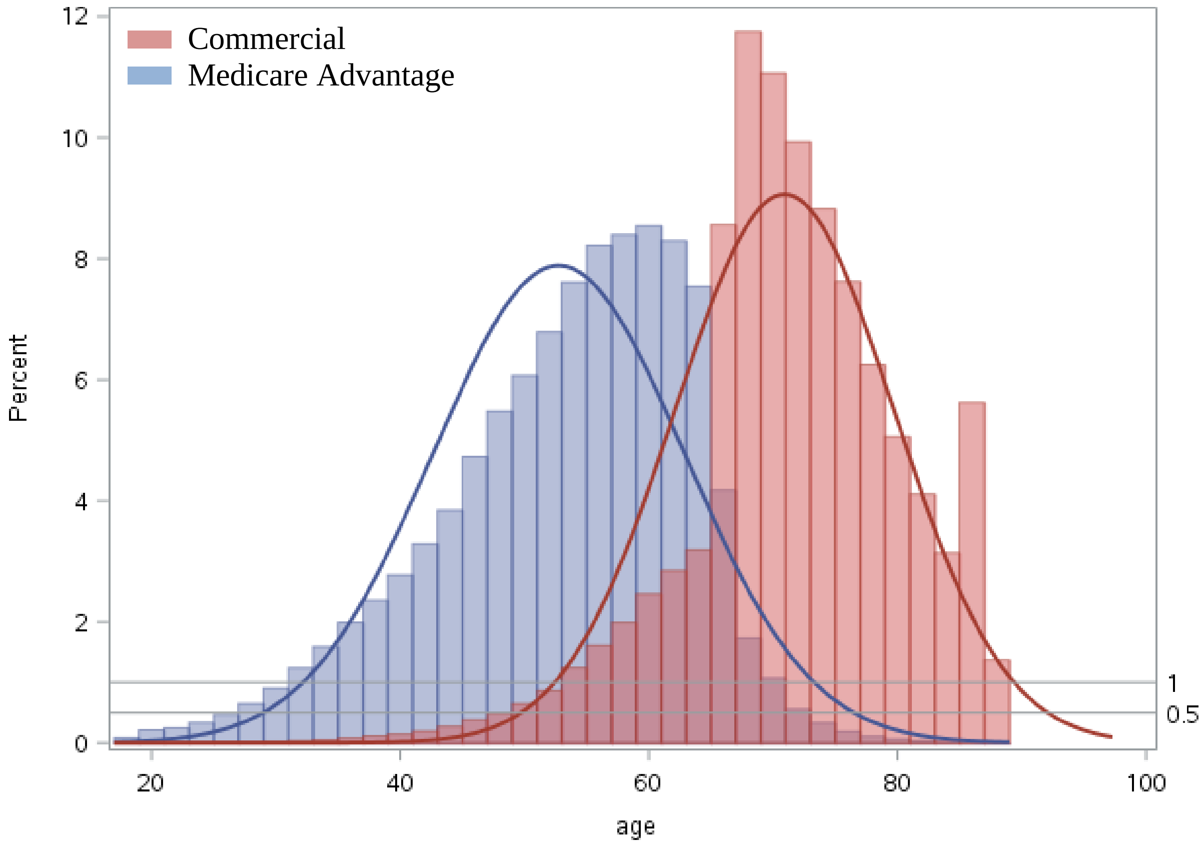

©2021 MCoy R et al. *JAMA Network Open*.
